# Functional Characteristics of Walnut Protein Fractions and Rutin Loading by Albumin

**DOI:** 10.3390/foods15122144

**Published:** 2026-06-14

**Authors:** Yue Wang, Xiang Li, Yu Zhou, Zilin Wang, Yuanli Wang, Fengyating Wu, Yang Tian, Liang Tao

**Affiliations:** 1 College of Food Science and Technology, Yunnan Agricultural University, Kunming 650201, China; 2 Engineering Research Center of Development and Utilization of Food and Drug Homologous Resources, Ministry of Education, Yunnan Agricultural University, Kunming 650201, China; 3 Yunnan Key Laboratory of Precision Nutrition and Personalized Food Manufacturing, Yunnan Agricultural University, Kunming 650201, China

**Keywords:** Yunnan deep-vein walnut, walnut protein fractions, functional characteristics, albumin, rutin

## Abstract

This study aimed to systematically compare the functional properties of the four major components (albumin, globulin, prolamin, and glutelin) of protein from Yunnan deep-veined walnuts to screen for protein-based carrier materials with good processing adaptability and the ability to efficiently encapsulate the active ingredient rutin. In addition, the binding and molecular interactions between the preferred protein and rutin were analyzed. The results indicated that albumin exhibited superior performance compared to the other three components in solubility, emulsifying properties, foaming properties, and gel properties, and demonstrated the strongest processing applicability. Further analysis revealed that albumin possessed an excellent amino acid composition (essential amino acid content accounting for 42.30%) and antioxidant activity (with the highest ABTS scavenging rate reaching 85.71 ± 0.26%), which indicated its considerable potential as a functional carrier. Loading rutin onto albumin yielded a walnut albumin–rutin complex (WA@Rut), which significantly enhanced the thermal stability of albumin (with the thermal denaturation temperature elevated to 108.72 °C) and the storage stability of rutin (66.16 ± 5.05% retention after 22 days of storage). Combined analyses of FT-IR spectroscopy, intrinsic fluorescence spectroscopy, molecular docking, and molecular dynamics simulations confirmed that rutin primarily bound to albumin via hydrogen bonding and electrostatic interactions, and formed a stable complex structure. SEM images revealed that the composite surface was smooth and exhibited a flake-like morphology. In conclusion, walnut albumin is a protein resource with significant functional potential in Yunnan deep-veined walnuts, and it exhibits strong processing applicability and enables efficient encapsulation and protection of active ingredients. This study provides novel strategies and theoretical foundations for the high-value utilization of walnut protein.

## 1. Introduction

Plant proteins, as essential ingredients in the food industry, have become a research hotspot for replacing animal proteins due to their significant advantages, including wide availability, low cost, high nutritional value, and environmental sustainability [1]. Identifying safe, healthy, and high-quality functional plant protein sources is an important goal for the future food industry [2]. Previous studies have demonstrated that plant proteins contribute to the stabilization of multilayer emulsions [3] as well as the formation of gels and thermoplastic films [4,5]. Moreover, plant proteins can be combined with other biopolymers for the targeted and sustained release of nutrients [6], the preparation of biodegradable films [7], and the enhancement of food quality and nutritional value [8]. Currently, legumes and cereals represent the primary sources of plant proteins. Among them, legume proteins such as soybean, chickpea, pea, lentil, and faba bean are considered to possess excellent commercial value owing to their favorable functional properties, including emulsifying, foaming, water- and oil-holding, and gelation capabilities [9]. In addition, rice bran protein has attracted considerable attention due to its high biological value [10]. In contrast, other types of plant proteins remain relatively underexplored. To meet the ever-growing demand for plant proteins, two major challenges currently persist: the limited variety of protein sources available for application and the inadequate applicability of existing plant proteins, both of which significantly constrain the broader utilization of plant proteins.

China is the world’s largest producer of walnuts. Yunnan, as a core region for walnut cultivation and production in China, ranks first nationwide in output. The primary cultivated variety is Yunnan deep-veined walnuts (*Juglans sigillata* Dode), and through long-term natural selection and artificial breeding, Yunnan has developed several superior cultivars, including Yangbi Pao walnut, Dayao Santai walnut, and Xixiang walnut. These cultivars are characterized by large nut size, thin shells, light-colored kernels, pure flavor, superior quality, and high yield [11]. Currently, walnut fruits are predominantly used for oil extraction. Walnut meal protein, a by-product of the walnut oil extraction process, accounts for approximately 40% of the dry weight of walnut meal and represents a high-quality source of plant protein [12]. However, the phytochemical compositions, including proteins, amino acids, and fatty acids, vary among different walnut varieties [13], resulting in considerable differences in their processing suitability and carrier capacity. In terms of composition, walnut protein primarily consists of glutelin, globulin, albumin, and prolamin, with each fraction possessing distinct functional properties. However, in-depth studies on individual protein components remain limited. Among the four proteins, glutelin has received the most attention, with the majority of research focusing on its modification and the exploration of functional peptides [14,15]. Nevertheless, in-depth research on individual protein fractions remains relatively scarce. Systematic characterization of the functional properties of protein fractions from Yunnan deep-veined walnuts is essential. Furthermore, exploring their potential applications in the food and industrial sectors represents a promising research direction to enhance added value and promote high-value utilization.

Rutin (also known as rutoside or quercetin-3-rutinoside) is a flavonol compound widely present in plants such as passionflower, buckwheat, tea, and apple. It possesses multiple pharmacological activities, including antioxidant, cytoprotective, vasoprotective, and anticancer effects, and has therefore attracted considerable attention [16]. However, rutin has poor water solubility and susceptibility to photodegradation, which limit its application in the functional food sector. Studies have shown that encapsulating rutin within plant proteins such as zein can effectively improve its solubility, dispersibility, and bioavailability [17]. As a highly promising carrier for the encapsulation and delivery of bioactive compounds, walnut protein has demonstrated great potential in enhancing the bioaccessibility of active ingredients such as lutein, β-carotene, and curcumin [18,19,20]. However, existing studies have predominantly focused on the encapsulation effects and endogenous polyphenol systems [21], while a systematic understanding of the binding mechanisms and structure–activity relationships between walnut protein and exogenous polyphenols is still lacking. This knowledge is of great scientific significance for the targeted improvement of protein functional properties and the expansion of application scenarios.

Currently, research on the refined separation and characterization of individual walnut protein fractions, their processing adaptability, and their application as carriers for bioactive compounds remains relatively scarce. In this study, the functional properties of the four protein fractions in walnut were first compared, and albumin, which exhibited the most comprehensive performance, was selected as the research target. The amino acid composition and antioxidant capacity of albumin were further characterized. On this basis, to enhance its loading and protective capacity for bioactive substances, albumin was employed as a carrier to achieve efficient loading of rutin. The structure–activity relationship of the walnut albumin–rutin complex (WA@Rut) was then elucidated through Fourier transform infrared (FT-IR) spectroscopy, molecular docking, molecular dynamics simulation and scanning electron microscopy (SEM). This study aims to improve the stability of rutin by constructing the WA@Rut system, thereby providing new insights into the rational design of plant protein-based carriers, as well as theoretical and technical references for the high-value utilization of walnut albumin.

## 2. Materials and Methods

### 2.1. Materials

Defatted walnut meal powder was provided by Yunnan Moore Farm Biotechnology Development Co., Ltd. (Chuxiong, Yunnan, China). Rutin (BR 95%) was purchased from Shanghai Yuanye Biotechnology Co., Ltd. (Shanghai, China). All other reagents used were of analytical grade.

### 2.2. Preparation of Four Walnut Protein Fractions

The four walnut protein fractions were extracted with slight modifications according to the method of Sze-Tao and Sathe [22]. Defatted walnut meal powder was dispersed in deionized water at a ratio of 1:15 (*w*/*v*) and stirred at room temperature for 2 h. The suspension was centrifuged (4000× *g*, 25 min, 4 °C), and the supernatant was collected as the albumin extract. The precipitate was extracted with 1 M NaCl (1:15, *w*/*v*) for 2 h, and the supernatant was collected as the globulin extract after centrifugation (4000× *g*, 25 min, 4 °C). The remaining precipitate was extracted with 70% ethanol (1:15, *w*/*v*) for 2 h, and the supernatant was collected as the prolamin extract after centrifugation (4000× *g*, 25 min, 4 °C). The final precipitate was extracted with 0.1 M NaOH (1:15, *w*/*v*) by stirring for 2 h, and the supernatant was collected as the glutelin extract. Each extraction step was repeated twice to ensure sufficient extraction of the protein fractions. The supernatants from different fractions were pooled, dialyzed at 4 °C for 48 h, and then freeze-dried. The resulting powders were stored at 4 °C for further use.

### 2.3. Functional Properties

#### 2.3.1. Protein Solubility

Protein solutions (1%, *w*/*v*) were prepared, and the pH was adjusted from 2.0 to 9.0 using 1 M HCl and NaOH. The solutions were centrifuged at 4500 rpm for 20 min at 4 °C, and the supernatants were collected. The protein content of the supernatants was determined using the Coomassie Brilliant Blue method, while the total protein content of the samples was determined by the Kjeldahl method. Protein solubility was expressed as the percentage of the protein content in the supernatant relative to the total protein content.

#### 2.3.2. Emulsifying Properties

A protein solution at a concentration of 1% (*w*/*v*) was prepared. A certain amount of walnut oil was added to achieve an oil volume fraction of 25% (*v*/*v*). The mixture was homogenized at 10,000 rpm for 3 min using a high-speed disperser at room temperature. The emulsion was diluted with 0.1% SDS (*v*/*v*, 100:1), mixed thoroughly, and the absorbance was measured at 500 nm. The absorbance of the solution after standing for 30 min was measured using the same method. The emulsifying activity index (EAI, m^2^/g) and emulsion stability index (ESI, min) were calculated according to the following formulas, respectively:

$$EAI(m^{2}/g)=\frac{2\times T\times A_{0}\times N}{c\times \left(1-\Phi \right)\times 10^{4}}$$

$$ESI(\min)=\frac{A_{0}}{A_{0}-A_{30}}\times 30$$

where T = 2.303, N is the dilution factor of the emulsion, c is the protein concentration in the aqueous protein solution (g/mL), Φ = 0.25 is the volume fraction of walnut oil in the original emulsion, and *A*_0_ and *A*_30_ are the absorbance values of the emulsion at 500 nm at 0 min and 30 min, respectively.

#### 2.3.3. Foaming Properties

Protein solutions (1%, *w*/*v*) were prepared and homogenized at 10,000 rpm for 3 min using a high-speed disperser. The foaming capacity (FC) and foam stability (FS) were calculated using the equations as follows:

$$\mathrm{FC}(\%)=\;\frac{V_{1}\;-\;V_{0}}{V_{0}}\times 100$$

$$\mathrm{FS}(\%)=\frac{V_{2}-V_{0}}{{V_{1}-V}_{0}}\times 100$$

where *V*_0_ is the volume of the solution before homogenization, *V*_1_ is the volume of the solution including foam immediately after homogenization, and *V*_2_ is the volume of the solution including foam after standing for 30 min.

#### 2.3.4. Rheological Properties

The four protein fractions were prepared as 10% (*w*/*v*) solutions in 10 mM phosphate buffer (pH 7.0) according to the method of Li et al. [23]. The solutions were loaded onto a DHR-2 rheometer for measurement, and silicone oil was applied around the edges of the plates to prevent moisture evaporation. The storage modulus (G′) and loss modulus (G″) were recorded throughout the measurement. The temperature scan parameters were as follows: the temperature was increased from 25 to 95 °C at a heating rate of 2 °C/min, held at 95 °C for 30 min for equilibration, and then cooled back to 25 °C at a cooling rate of 2 °C/min. During the temperature scan, the frequency was set at 1 Hz with a strain of 1%. After the temperature scan, a frequency sweep was performed at 25 °C with a strain of 1% over a frequency range of 0.1–10 Hz for 20 min, during which G′ and G″ were recorded.

### 2.4. Characterization of Albumin

#### 2.4.1. SDS-PAGE

Freeze-dried albumin was subjected to electrophoresis under reducing conditions using a 12% separating gel and a 4% stacking gel. A 1% (*w*/*v*) protein solution was mixed with the loading buffer at a volume ratio of 1:1. The mixture was heated in a metal bath for 10 min and then centrifuged. A total of 15 μL of the supernatant and 10 μL of molecular weight protein markers (10–180 kDa) were loaded into each lane of the gel. After electrophoresis, the gel was stained with Coomassie Brilliant Blue staining solution for 5 h, followed by destaining in a solution containing 5% ethanol and 10% glacial acetic acid. The gel was then imaged using a gel imaging system.

#### 2.4.2. Amino Acid Composition

The four protein samples were derivatized and subsequently quantified by high-performance liquid chromatography (HPLC) using an AccQ Tag (Water Corporation, Milford, MA, USA) amino acid analysis column (150 × 4.6 mm, 5 μm).

#### 2.4.3. Antioxidant Capacity

The assay was performed with slight modifications according to the method described by Razali et al. [24]. Briefly, 4 mg of DPPH was dissolved in 100 mL of absolute ethanol to prepare a 0.1 mM DPPH ethanolic solution, which was stored in a sealed brown bottle at 4 °C in the dark. A 0.5 mL aliquot of the sample solution at various concentrations was mixed with 0.5 mL of the 0.1 mM DPPH ethanolic solution in a 2 mL centrifuge tube. The mixture was allowed to react in the dark for 50 min and then centrifuged (10 min, 4000 r/min). The absorbance was measured at 517 nm, and the radical scavenging activity was calculated using the following equation:

$$DPPH\;radical\;scavenging\;activity(\%)=\frac{A_{c}-(A_{s}-A_{0})}{A_{c}}\times 100$$

where *A*_0_ is the absorbance of the blank control, in which 0.5 mL of absolute ethanol was used to replace the 0.1 mM DPPH solution; *A*_c_ is the absorbance of the control, containing 0.5 mL of absolute ethanol and 0.5 mL of 0.1 mM DPPH ethanolic solution; and *A*_s_ is the absorbance of the sample, containing 0.5 mL of the sample solution and 0.5 mL of 0.1 mM DPPH ethanolic solution.

A mixture of 2.45 mM potassium persulfate and 7 mM ABTS stock solution was prepared at a ratio of 1:1 (*v*/*v*) and allowed to react in the dark for 12–16 h. The resulting ABTS radical cation solution was then diluted with 5 mM phosphate-buffered saline (PBS, pH 7.4) until the absorbance reached 0.7–0.8 at 734 nm. Subsequently, 0.5 mL of the sample solution at various concentrations was mixed with 0.5 mL of the diluted ABTS radical working solution at a ratio of 1:1 (*v*/*v*) and incubated in the dark at room temperature for 10 min. The radical scavenging activity was calculated using the equation as follows:

$$\mathrm{ABTS}\;\mathrm{radical}\;\mathrm{scavenging}\;\mathrm{activity}(\%)=\frac{A_{0}{-A}_{s}}{A_{0}}\times 100$$

where *A*_s_ is the absorbance of the sample and *A*_0_ is the absorbance of the blank control.

### 2.5. Preparation and Binding Characteristics Analysis of WA@Rut

#### 2.5.1. Preparation of WA@Rut

Walnut albumin was dispersed in distilled water to prepare a 1% (*w*/*v*) solution, and rutin was dissolved in a 20% ethanol–water solution (*v*/*v*) to obtain a 1 mg/mL solution. The two solutions were then mixed at a specific volume ratio and magnetically stirred for 2 h. The mixture was then heated at 70 °C for 20 min with continuous stirring in the dark, followed by immediate cooling in an ice bath. Excess ethanol was removed by rotary evaporation, and the sample was freeze-dried for subsequent use, yielding WA@Rut.

#### 2.5.2. Stability Analysis of WA@Rut

The samples were sealed and stored in the dark at 60 °C for 22 days. Samples were collected every 2 days, and the absorbance was measured at 510 nm using the NaNO_2_-Al(NO_3_)_3_-NaOH colorimetric method to determine the rutin content. The rutin retention rate was calculated according to the following equation:

$$Retention\;rate(\%)=\frac{W_{1}-W_{2}}{W_{1}}\times 100$$

where *W*_1_ is the rutin content at the beginning of storage, and *W*_2_ is the rutin content during storage.

#### 2.5.3. Differential Scanning Calorimetry (DSC)

The thermal properties of pure rutin, albumin, and WA@Rut were determined using a differential scanning calorimeter with a heating rate of 10 °C/min over a temperature range of 20–200 °C.

#### 2.5.4. FT-IR

Samples were mixed with potassium bromide (KBr) and pressed into transparent pellets using a hydraulic press. Spectra were recorded with a Nicolet IS5 spectrometer (ThermoFisher Scientific, Waltham, MA, USA) over the range of 4000–400 cm^−1^ with 32 scans at a resolution of 4 cm^−1^.

#### 2.5.5. Intrinsic Fluorescence Spectroscopy

The intrinsic fluorescence spectra of the proteins were scanned using a Synergy H1m microplate reader (BioTek Instruments, Inc., Winooski, VT, USA) with an emission wavelength range of 300–500 nm and an excitation wavelength of 280 nm.

#### 2.5.6. Molecular Docking

The protein structure used for docking was downloaded from the UniProt database (accession number: P93198). The 3D structure of rutin was generated by importing its SMILES into Maestro and subsequently energy-minimized under the OPLS4 force field. Molecular docking was performed using AutoDock Vina 1.2.5. Prior to docking, all receptor proteins were processed using PyMOL 2.5, including the removal of water molecules, salt ions, and small molecules. The docking box was then defined with the centroid of the co-crystallized ligand set as the center and a box size of 35 Å. Additionally, all processed small molecules and receptor proteins were converted to the PDBQT format required by AutoDock Vina 1.2.5 using ADFRsuite 1.0. During docking, the exhaustiveness of the global search was set to 32, while all other parameters were kept at their default values.

#### 2.5.7. Molecular Dynamics Simulation

Based on the docked complex, all-atom molecular dynamics simulations were performed using AMBER 24 [25]. Partial charges for the ligand were calculated using the AM1-BCC method via the antechamber module. The protein and ligand were described using the ff14SB and GAFF2 force fields, respectively. Hydrogen atoms were added using the LEaP module, and a truncated octahedral TIP3P water box was constructed with a 10 Å boundary from the solute. Na^+^/Cl^−^ ions were added to neutralize the system charge. Energy minimization was carried out in two stages: 2500 steps of steepest descent followed by 2500 steps of conjugate gradient. Subsequently, the system was gradually heated from 0 K to 298.15 K over 200 ps under the NVT ensemble, followed by 500 ps of NVT equilibration to allow full solvent relaxation, and then 500 ps of NPT equilibration. Finally, a 100 ns NPT production run was conducted with a non-bonded cutoff of 10 Å, long-range electrostatics treated by the particle mesh Ewald (PME) method, bonds involving hydrogen constrained using the SHAKE algorithm, temperature controlled by the Langevin thermostat (collision frequency γ = 2 ps^−1^), pressure maintained at 1 atm, and an integration time step of 2 fs. Trajectories were saved every 10 ps for subsequent analysis.

#### 2.5.8. SEM

The morphology of the samples was observed using a ZEISS Sigma 300 scanning electron microscope (Carl Zeiss Microscopy GmbH, Jena, Germany).

### 2.6. Statistical Analysis

All experiments were conducted in triplicate, and data from three parallel measurements were expressed as mean ± standard deviation. Statistical analysis was performed using SPSS 19.0 software. One-way analysis of variance (ANOVA) was applied, and mean comparisons were carried out using Tukey’s HSD test. A *p* < 0.05 was considered statistically significant.

## 3. Results and Analysis

### 3.1. Comparison of Properties of Walnut Protein Fractions

#### 3.1.1. Protein Content

**Figure 1:**
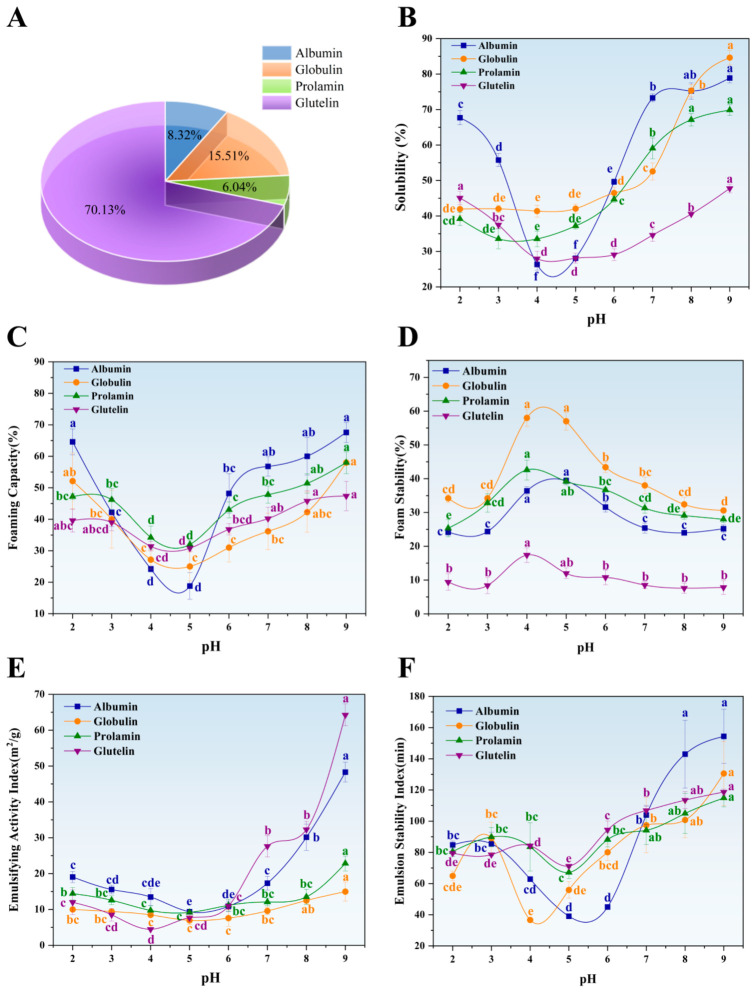
Properties of walnut protein components. (**A**) Protein composition. (**B**) Solubility. (**C**,**D**) Foaming capacity and foaming stability. (**E**,**F**) Emulsifying capacity and emulsifying stability. Groups designated with different letters differ significantly (*p* < 0.05).

As shown in Figure 1A, the content of each protein fraction in walnut protein followed the order of glutelin > globulin > albumin > prolamin, which is consistent with the findings reported by Mao et al. [26]. Currently, research on walnut protein fractions remains relatively limited, with the majority of attention focused on glutelin, as it accounts for over 70% of the total walnut protein, and thus, exerts the greatest influence on the overall characteristics of the protein profile. However, glutelin exhibits relatively poor functional properties. To address this, existing studies have predominantly employed various modification approaches, including enzymatic hydrolysis [27], acylation and glycosylation [28], and atmospheric cold plasma treatment [14], to optimize its functional characteristics, thereby improving the processing suitability and application scope of the entire walnut protein. Nevertheless, investigating the refined applications of individual protein fractions also represents a critical direction for achieving the precise utilization of walnut protein. Therefore, although the other protein fractions constitute a relatively small proportion, they may possess superior processing characteristics and application potential, and thus remain of significant research value. Conducting detailed studies on different protein fractions holds considerable practical significance.

#### 3.1.2. Solubility

As shown in Figure 1B, the solubility of albumin, globulin, prolamin, and glutelin under different pH conditions (2.0–9.0) exhibited a typical U-shaped curve. Similar to most plant proteins, the four walnut protein fractions displayed the lowest solubility near their isoelectric points (pI), all of which were located at approximately pH 4.0. At this point, the electrostatic repulsion between protein molecules was weakened, the protein–water interaction was reduced, and intermolecular hydrophobic interactions were enhanced, thereby promoting protein aggregation and precipitation. On either side of the pI, proteins carried net positive or negative charges, resulting in increased electrostatic repulsion and consequently higher solubility [29]. Under neutral conditions, the solubility of albumin reached 73.29 ± 1.13%, which was significantly higher than that of glutelin (34.60 ± 1.76%) (*p* < 0.001). In a weakly alkaline environment (pH 9.0), the ionization of carboxyl groups and the deprotonation of amino groups generated negatively charged species, which improved protein–solvent interactions and thereby enhanced protein solubility [30]. The solubility of globulin and albumin reached 84.63 ± 2.06% and 78.88 ± 1.40%, respectively. In summary, albumin and globulin exhibited good solubility under neutral and weakly alkaline conditions, which holds positive application value for improving the processing suitability and functional properties of poorly soluble fractions and for integrating raw material systems. Therefore, they are suitable for product development in plant-based milk, neutral sauces, baked goods, and other food applications.

#### 3.1.3. Foaming Capacity and Foaming Stability

Protein foaming properties are primarily determined by FC and FS. FC is related to protein hydrophobicity and the interfacial area that can be formed, whereas FS reflects the ability of foam to resist external stress [31]. As illustrated in Figure 1C,D, the FC and FS of the four walnut protein fractions exhibited opposite trends with varying pH: FC was lowest while FS was highest near the pI; as pH deviated from the pI, FC gradually increased while FS correspondingly decreased. Foaming performance is closely associated with protein solubility. Near the pI, protein solubility decreases and aggregation becomes pronounced, resulting in a reduced amount of soluble protein available for foam formation and thus a lower FC [32]. Conversely, the decreased solubility facilitates protein adsorption and aggregation at the interface, forming a rigid network structure that serves as a stabilizing skeleton for the foam film, thereby enhancing FS [33]. When pH deviates from the pI, electrostatic repulsion among protein molecules increases while hydrophobic interactions weaken, improving molecular flexibility and surface adsorption rate. These changes favor rapid encapsulation of air and foam formation [34]. In terms of compositional differences, glutelin and globulin exhibited relatively poor foaming performance, which may be attributed to their larger molecular size and globular conformation, leading to slower interfacial adsorption rates and consequently lower FC [35]. Overall, walnut albumin and prolamin demonstrated superior FC and FS under weakly acidic, weakly alkaline, and neutral conditions. The high flexibility of albumin may account for its better foaming properties compared to the other fractions [34]. These protein fractions are suitable for application in baking, aerated beverages, dairy products, meal-replacement foamed foods, and other processing technologies. Furthermore, protein foaming creates an adsorption layer at the gas–liquid interface, enabling the encapsulation of polyphenols within the foam interfacial film and the protein network structure. This significantly enhances the solubility and dispersion uniformity of polyphenols in aqueous systems, isolates them from the external environment, and reduces oxidative degradation caused by direct exposure. Additionally, the synergistic antioxidant effect between proteins and polyphenols further improves product quality.

#### 3.1.4. Emulsification and Emulsified Stability

The EAI reflects the ability of proteins to adsorb at the oil–water interface and form emulsions, whereas the ESI indicates their capacity to maintain emulsion stability [36]. As shown in Figure 1E,F, both the EAI and ESI of the four walnut protein fractions exhibited a trend of first decreasing and then increasing with rising pH, reaching their lowest values near the pI, where EAI and ESI were the poorest. This phenomenon is closely related to protein solubility. Near the pI, the reduced solubility hinders effective protein adsorption at the oil–water interface, preventing sufficient reduction in interfacial tension and the formation of a stable interfacial film, thereby impairing emulsifying performance [37]. Under acidic conditions, albumin and prolamin showed slightly higher EAI values than globulin and glutelin, which may be attributed to differences in molecular structure and conformational flexibility among the protein fractions [38]. Notably, under alkaline and neutral conditions, despite the relatively low solubility of glutelin, its EAI remained comparatively high, which may be associated with its hydrophobic interactions [39]. Studies have demonstrated that a greater number of hydrophobic groups on proteins favors a more pronounced reduction in interfacial tension, thereby enhancing emulsifying performance [40]. Under neutral conditions, both albumin and glutelin exhibited favorable emulsifying properties, indicating their potential as food emulsifiers. They can be applied in a wide range of food systems, including salad dressings, plant-based creams, spreadable sauces, beverage emulsion systems, and processed meat alternatives, where they perform emulsification, stabilization, and texture improvement functions. Excellent EAI and ESI confer enhanced resistance to pH variations, salt tolerance, and high-shear processing, with minimal risk of emulsion breakdown or flocculation during manufacturing. This is of positive significance for maintaining the stability of complex food component systems and is well-suited for industrial continuous processing.

#### 3.1.5. Gelation Properties

**Figure 2:**
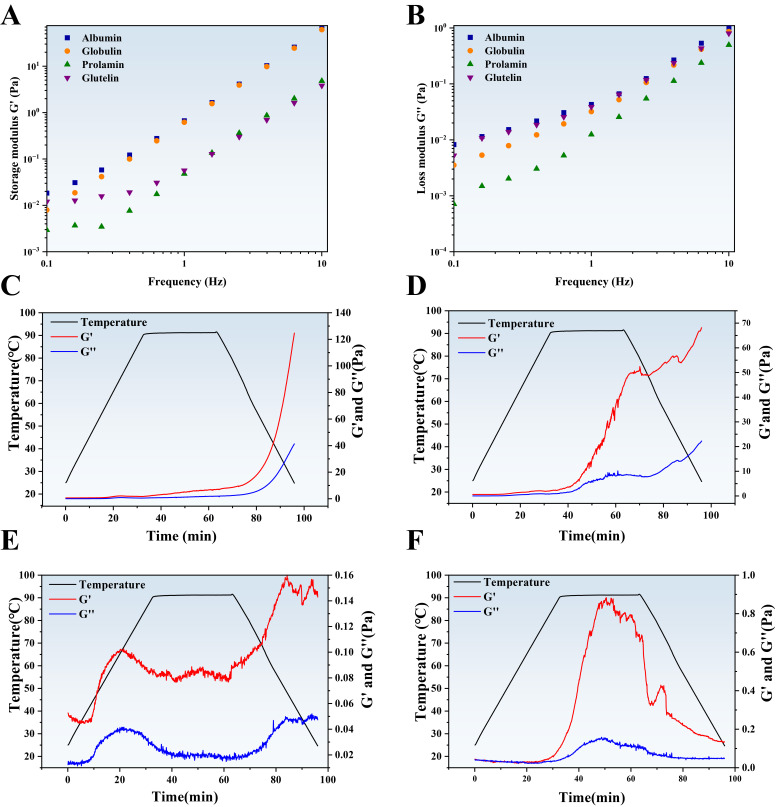
Rheological properties of walnut protein components. (**A**) G′ of the four proteins as a function of frequency. (**B**) G″ of the four proteins as a function of frequency. (**C**–**F**) Effects of temperature and time on the G′ and G″ of albumin, globulin, prolamin, and glutelin.

In rheological analysis, the G′ represents the solid-like characteristic of a material and its ability to maintain elastic deformability, whereas the (G″ reflects the liquid-like characteristic and the viscous nature of the sample. Tan δ = (G″/G′) is used to characterize the viscoelastic behavior of the material, with Tan δ < 1 indicating that the sample exhibits predominantly elastic, solid-like properties [41]. As shown in Figure 2A,B, both G′ and G″ increased with increasing frequency, demonstrating strong frequency dependence. For all four protein fractions, G′ was consistently greater than G″ and Tan δ remained below 1, indicating that these proteins behaved predominantly elastically and exhibited solid-like mechanical behavior. Notably, at frequencies above 1 Hz, the four protein fractions exhibited significant differences in G′, with the following order: albumin > globulin > prolamin > glutelin. Specifically, at 10 Hz, the G′ values of albumin and globulin were approximately 13 and 16 times higher than those of prolamin and glutelin, respectively, suggesting that the gel networks formed by albumin and globulin possessed higher elasticity and a more compact structure [42]. From the perspective of aggregation behavior, albumin and globulin exhibited better water dispersibility and were more prone to forming small, uniformly distributed protein aggregates, which facilitated the construction of more thoroughly cross-linked and densely structured gel networks, thereby resulting in higher G′ values [43].

Figure 2C–F illustrate the variations in G′ and G″ of the four walnut protein fractions during heating (25–90 °C), holding (90 °C), and cooling. Throughout the entire thermal treatment, the G′ values of albumin and globulin were consistently and significantly higher than those of prolamin and glutelin, indicating that the former two fractions possessed a stronger capacity for heat-induced gel formation. During the initial heating stage (25–90 °C), both G′ and G″ of albumin and globulin increased gradually with rising temperature, with G′ remaining consistently greater than G″, suggesting that protein molecules progressively unfolded and aggregated upon heating, initiating the formation of a preliminary elastic network structure. Notably, globulin exhibited a sharp increase in G′ during the holding stage (90 °C), whereas albumin showed a marked rise in G′ during the cooling phase. The continuous increase in both G′ and G″ observed during the holding and cooling stages may be attributed to the progressive incorporation of an increasing number of protein molecules into the protein network during the holding period, thereby further strengthening the gel network. During the cooling phase, the enhanced intermolecular and intramolecular hydrogen-bonding interactions among protein molecules further elevated G′, reinforcing the gel network structures of albumin and globulin [44].

In contrast, prolamin and glutelin exhibited distinct rheological characteristics. For prolamin, both G′ and G″ gradually increased during the initial heating stage, followed by a decrease in G′ at approximately 65 °C, and a subsequent rise during the cooling phase. This behavior may be attributed to the re-aggregation of denatured protein molecules through hydrogen bonding and hydrophobic interactions [45]. For glutelin, both G′ and G″ increased gradually during the initial heating stage and rose rapidly during the holding stage; however, they declined during the cooling phase. This may be because glutelin formed large aggregates during heating, resulting in an enlarged gel network pore size, reduced cross-linking density, and consequently inferior mechanical properties [43]. In summary, albumin and globulin demonstrated significantly superior gelation performance compared to prolamin and glutelin, owing to their higher storage modulus and stronger capacity for heat-induced gel formation. In product processing, the three-dimensional network structure of protein gels can entrap bioactive components and effectively mask undesirable sensory attributes such as bitterness and astringency, thereby improving the sensory properties of the final product. The results indicate that albumin exhibits excellent solubility and foaming capacity under neutral and weakly alkaline conditions, along with significant gelation potential. Although its emulsifying capacity is not the strongest, its emulsion stability under the same conditions is superior. Overall, albumin demonstrates superior functional properties compared to the other three proteins and possesses greater processing potential; therefore, subsequent research will focus on albumin.

### 3.2. Albumin Characteristics

#### 3.2.1. Molecular Weight Distribution

**Figure 3:**
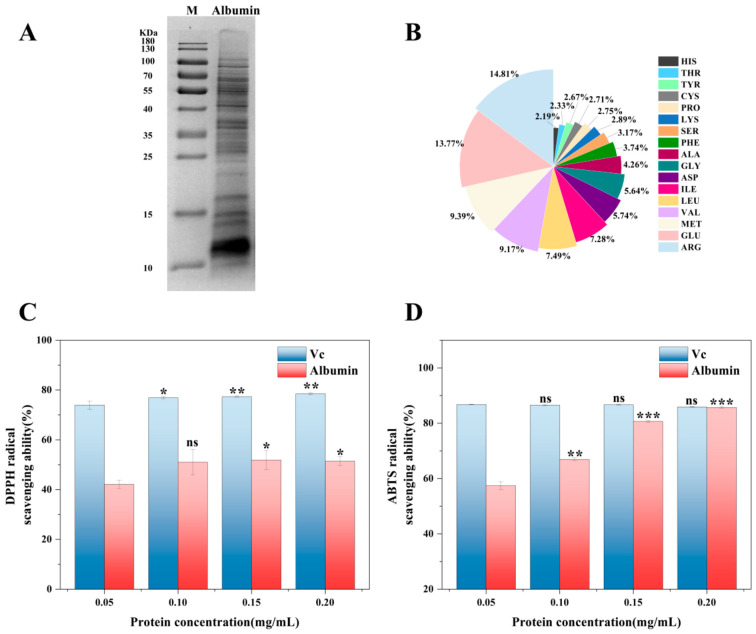
Characterization of albumin. (**A**) SDS-PAGE (Lane M: protein marker; Lane Albumin: albumin). (**B**) Amino acid composition. (**C**) DPPH radical scavenging rate. (**D**) ABTS radical scavenging rate. Vc stands for ascorbic acid. Statistical significance was determined as follows: ns for no significant difference,* for *p* < 0.05, ** for *p* < 0.01, and *** for *p* < 0.001.

As shown in Figure 3A, the albumin subunits exhibited a broad distribution across the molecular weight range of 10–100 kDa, with relatively uniform band patterns observed throughout this interval. Notably, more intense bands were detected within the 10–15 kDa range, which is consistent with the findings of Sze-Tao and Sathe [22]. The small-molecular-weight subunit component within the 10–15 kDa range may account for the superior functional properties of albumin. Previous studies have demonstrated that low-molecular-weight peptides generally exhibit better interfacial properties than high-molecular-weight proteins, and thus, their enrichment contributes to improved emulsion quality [46]. Meanwhile, the presence of low-molecular-weight peptides has also been confirmed to enhance gel network formation [47].

#### 3.2.2. Amino Acid Composition

The amino acid composition analysis results are presented in Figure 3B. The most abundant amino acid in albumin was arginine, followed by glutamic acid. As a conditionally essential amino acid, arginine has been reported to reduce cholesterol levels, thereby benefiting cardiovascular health [48]. Furthermore, the essential amino acid content accounted for 42.30% of the total amino acids. According to the FAO/WHO/UNU standard [49], a protein is considered to be of high quality when the proportion of essential amino acids to total amino acids reaches or exceeds 35%. Collectively, walnut albumin represents a high-quality plant protein with considerable nutritional value. It serves as an excellent source of dietary amino acids and holds promising potential for application in the development of functional foods.

#### 3.2.3. Antioxidant Capacity

As illustrated in Figure 3C,D, within the concentration range of 0–0.2 mg/mL, the DPPH radical scavenging rate increased with increasing sample concentration, reaching a maximum of 51.90 ± 3.83%. Notably, albumin exhibited a more pronounced scavenging effect on ABTS radicals, achieving a scavenging rate of 85.71 ± 0.26% at a concentration of 0.2 mg/mL. Substances with strong antioxidant properties can be widely utilized in the development of functional foods, cosmetics, and pharmaceuticals, and may help prevent and reduce the risk of various diseases. Given its favorable antioxidant activity, albumin demonstrates considerable potential for development as a natural antioxidant.

### 3.3. Characteristics and Binding Interaction Analysis of WA@Rut

#### 3.3.1. Stability

**Figure 4:**
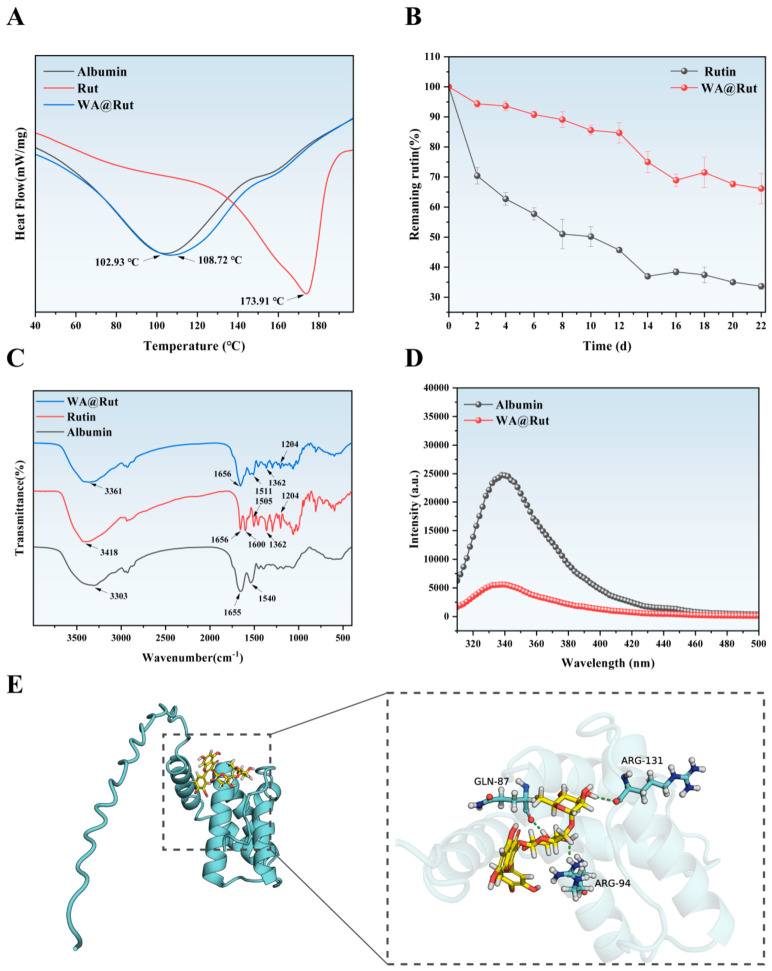
Characterization of the complex. (**A**) DSC analysis. (**B**) Storage stability. (**C**) FT-IR spectra. (**D**) Intrinsic fluorescence spectra. (**E**) Molecular docking (**left**: overall view; **right**: close-up view; yellow and red sticks represent rutin, blue cartoon represents the protein, and green dashed lines indicate hydrogen-bond interactions).

Stability is a critical evaluation parameter for food products during production, processing, transportation, and storage. The thermal stability and storage stability of the complexes were investigated. DSC thermograms (Figure 4A) revealed that the characteristic melting peak of rutin at 173.91 °C disappeared in the WA@Rut complex, indicating that rutin no longer existed in a free crystalline state but was instead embedded into the complex at the molecular level [50]. Meanwhile, the endothermic peak of WA@Rut shifted from 102.93 °C to 108.72 °C, suggesting that the binding of rutin exerted a certain enhancing effect on the thermal stability of albumin. The storage stability results are presented in Figure 4B. The rutin content exhibited a rapid decline with increasing storage time. After storage at 60 °C for 10 d, the rutin content decreased to 50.18 ± 3.29%, and further declined to 33.62 ± 0.94% by 22 d. Upon encapsulation with walnut albumin, the stability of rutin was significantly improved, with residual contents of 85.56 ± 1.58% at 10 d and 66.16 ± 5.05% at 22 d. These results indicate that walnut albumin provides effective protection for rutin. The enhanced stability conferred by walnut albumin facilitates the retention of bioactive components, which is more favorable for product processing and storage. This finding supports the development of high-stability, high-bioavailability rutin-enriched functional plant-based protein products.

#### 3.3.2. FT-IR

As shown in Figure 4C, the absorption peak of albumin at 3303 cm^−1^ was attributed to the N-H stretching vibration. In WA@Rut, this peak shifted to a higher wavenumber at 3361 cm^−1^, suggesting the formation of hydrogen-bond interactions between the N–H groups of albumins and rutin, which led to an increase in wavenumber [17,51]. The characteristic peaks of rutin were identified at 3418 cm^−1^ (O–H), 1656 cm^−1^ (C=O), 1505 cm^−1^ (C=C), 1362 cm^−1^ (C–O), and 1204 cm^−1^ (C–O–C), which were consistent with previously reported values [52]. In the WA@Rut complex, within the range of 1600–532 cm^−1^, multiple characteristic peaks of rutin either disappeared or were significantly attenuated, with only a few peaks, such as those at 1362 and 1204 cm^−1^, remaining as weak bands with slight shifts. The suppression of these characteristic peaks indicates that the molecular vibrations of rutin were restricted, suggesting the presence of interfacial interactions or encapsulation [53]. Additionally, a shift in the characteristic absorption peak of the amide II band was observed, suggesting possible electrostatic interactions between the active compound and the carrier protein [54], which is consistent with the findings reported by Khan et al. [55]. The absence of new peaks in the complex indicated that the encapsulation process was dominated by non-covalent interactions such as hydrogen bonding and electrostatic forces, rather than covalent reactions. These results suggest that rutin binds to albumin primarily through hydrogen bonding and electrostatic interactions, which may be the main reason for the enhanced stability.

#### 3.3.3. Intrinsic Fluorescence

As shown in Figure 4D, under excitation at 280 nm, albumin exhibited a typical intrinsic fluorescence emission peak at 340 nm. Upon the addition of rutin, no significant shift in the emission peak position was observed, indicating that the polarity of the microenvironment surrounding tyrosine residues remained largely unchanged [56]. Fluorescence quenching has been proven to be an effective method for evaluating the binding status between proteins and other molecules [57]. The significant decrease in fluorescence intensity of albumin was presumably attributed to the interaction between rutin and the tyrosine residues of albumin, which led to the quenching of more tyrosine fluorescence within the albumin structure, thereby resulting in a reduction in fluorescence intensity. This indicates the formation of a complex between the two molecules.

#### 3.3.4. Molecular Docking Results

The binding mode of walnut albumin with rutin obtained from molecular docking is shown in Figure 4E. Rutin forms hydrogen bonds with GLN-87, ARG-94, and ARG-131 of walnut albumin. The binding mode is characterized by a slight outward convexity into the internal cavity, with a docking score of −6.993 kcal/mol, indicating a strong affinity between rutin and walnut albumin. Hydrogen bonding serves as the primary driving force for the formation of the stable complex. These binding characteristics are consistent with the protein–polyphenol interaction patterns reported by Baruah et al. [58].

#### 3.3.5. Molecular Dynamics Simulation Results

**Figure 5:**
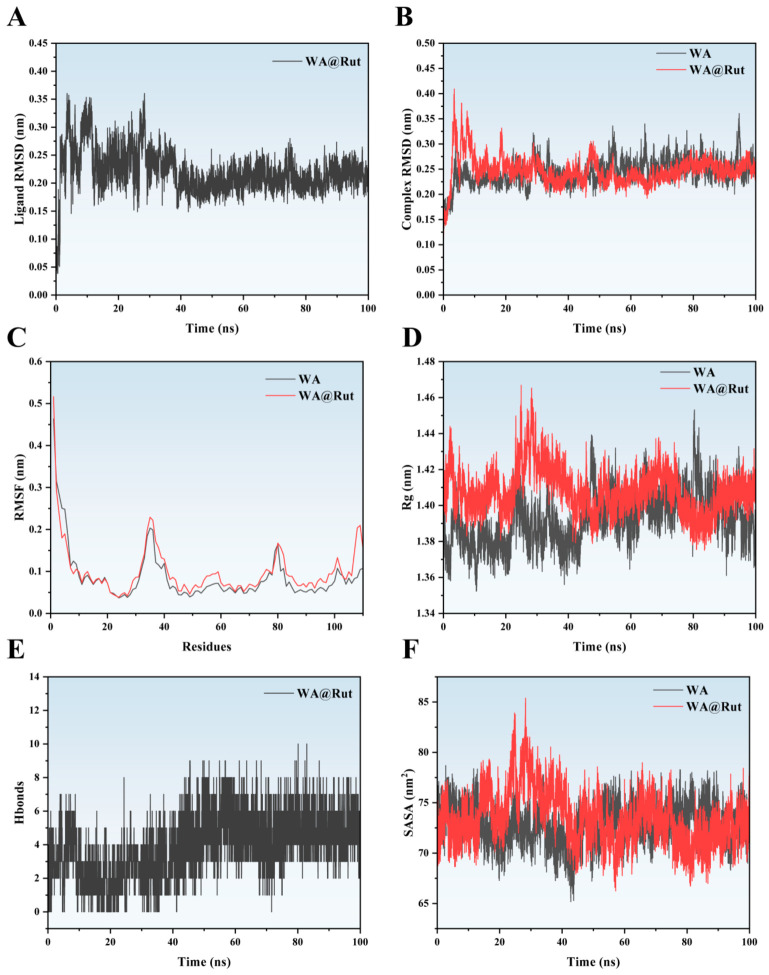
Molecular dynamics simulation results. (**A**) RMSD of the ligand during the simulation. (**B**) RMSD of the complex. (**C**) RMSF of the protein. (**D**) Rg of the complex. (**E**) Number of hydrogen bonds in the complex. (**F**) SASA of the complex as a function of simulation time.

To evaluate the conformational stability and dynamic behavior of rutin upon binding to walnut albumin, a 100 ns molecular dynamics simulation was performed on walnut albumin and the complex (WA@Rut) (Figure 5A–F). As shown in Figure 5A, the ligand root-mean-square deviation (RMSD) rapidly adjusted during the initial phase of the simulation and subsequently stabilized, maintaining only minor fluctuations in the later stages. This indicates that rutin can sustain a relatively stable binding pose within the binding pocket without significant drift or dissociation. Figure 5B shows that the overall RMSD profiles of the complex and the free protein were comparable, both reaching equilibrium within a short time and remaining stable thereafter. After 50 ns, the RMSD of albumin stabilized at 0.2–0.36 nm, while that of the complex stabilized at 0.19–0.29 nm, suggesting that ligand binding did not disrupt the overall protein conformation and that the system stability was modestly enhanced [58]. Root-mean-square fluctuation (RMSF) is commonly used to identify residue-level fluctuations and serves as an important indicator for interpreting protein activity and conformational stability [59]. As illustrated in Figure 5C, the RMSF profiles of walnut albumin and WA@Rut exhibited an overall consistent trend, with certain regions showing slightly higher fluctuations compared to the native albumin. This suggests that rutin has a limited effect on the overall flexibility of the protein but may modulate the dynamics of specific local regions. The radius of gyration (Rg) of the complex was used to characterize the compactness of binding; a lower Rg value indicates a more compact complex structure. As shown in Figure 5D, during the initial phase up to approximately 40 ns, the Rg value of the WA@Rut complex was relatively higher, indicating that rutin binding reduced the compactness of the protein and induced a more extended conformation. This phenomenon may be attributed to the insertion of rutin into albumin, which promotes outward expansion of the protein [60]. As the simulation progressed, both the protein and the corresponding complex reached equilibrium and remained stable, with Rg values approaching those of the free protein, indicating that the overall folding compactness of the protein did not undergo significant alteration. Figure 5E shows that multiple hydrogen bonds were continuously formed throughout the simulation and maintained at a relatively high frequency in the later stages, suggesting that the hydrogen-bond network serves as a key driving force for stabilizing ligand binding, which is consistent with the molecular docking and FT-IR results. The solvent-accessible surface area (SASA) curve shown in Figure 5F remained generally stable, with WA@Rut exhibiting slightly higher values than those of albumin overall, possibly indicating a conformational expansion of the protein upon rutin binding.

Overall, rutin forms a stable complex with walnut albumin through sustained non-covalent interactions. Additionally, the MM/GBSA results (Table 1) indicated that the binding energy of WA@Rut was −28.92 ± 3.26 kcal/mol. The negative value indicates binding affinity between the molecule and the target protein, with lower values corresponding to stronger binding, suggesting that albumin and rutin possess spontaneous binding capability. Energy decomposition analysis revealed that the dominant contributions to binding were electrostatic and van der Waals energies.

#### 3.3.6. Microstructure

**Figure 6:**
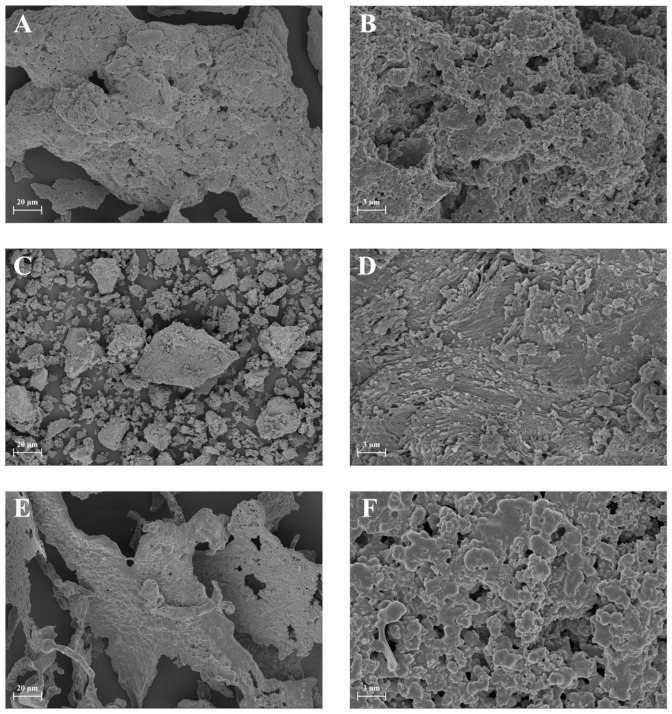
SEM images of the complex. (**A**,**B**) Albumin (magnification: 500×, 3500×). (**C**,**D**) Rutin (magnification: 500×, 3500×). (**E**,**F**) WA@Rut (magnification: 500×, 3500×).

Figure 6A–F present the micro-morphologies of albumin, rutin, and WA@RUT at different magnification levels. As shown in the figure, albumin exhibited an irregular block-like structure with a rough surface, whereas rutin appeared as loose, fragmented blocks with needle-like crystals observable at a magnification of 3500× [61]. In contrast, WA@RUT displays a flake-like structure, and at 3500× magnification, the surface of the complex tends to be smoother, with evident adhesion between molecules. Notably, the needle-like crystalline features of free rutin observed at 3500× magnification completely disappeared in the WA@Rut complex, indicating that rutin molecules were successfully encapsulated within the albumin matrix. This is consistent with the DSC results, which showed a transition from a crystalline to an amorphous state. The obvious adhesion between molecules, forming larger particles, may result from the aggregation of smaller nanoparticles [62]. This morphological feature is similar to that of the quercetin–zein complex reported by Yong et al. [63]. The intermolecular adhesion may also be attributed to hydrogen bonding formed between the particles and water molecules during solvent evaporation and freeze-drying [17].

## 4. Conclusions

Walnut protein primarily consists of four fractions: albumin, globulin, prolamin, and glutelin. This study systematically evaluated the processing suitability of each fraction, revealing significant differences in their functional properties. Particular attention was given to the albumin fraction, which, despite its relatively low proportion, exhibited excellent solubility, emulsifying capacity, foaming ability, and gelation properties, demonstrating considerable potential for product development. A WA@Rut complex was successfully prepared using walnut albumin as a carrier to load rutin, and the carrier significantly enhanced the stability of rutin. The binding between albumin and rutin is mediated by hydrogen bonding and electrostatic interactions, making it a natural delivery carrier. However, the specific primary amino acid sequence of albumin and its systematic identification at the proteomics level remain lacking. Future studies should employ proteomics technologies to elucidate the amino acid sequence and subunit composition of albumin, thereby further supporting the reliability of structure–function relationship studies such as molecular docking. Meanwhile, this study has not yet deeply explored the release behavior and mechanism of rutin from the WA@Rut complex during gastrointestinal digestion. Future studies could systematically examine the gastrointestinal digestion, absorption stability, and release kinetics of this delivery system, evaluate its *in vivo* bioavailability, and elucidate its health-regulatory effects and associated molecular mechanisms, thereby providing a comprehensive assessment of the delivery performance and application value of walnut albumin.

## Figures and Tables

**Table 1:** Binding free energies and individual energy components predicted by the MM/GBSA method (kcal/mol).

System Name	WA@Rut
Δ*E*_vdw_	−38.16 ± 3.80
Δ*E*_elec_	−72.18 ± 7.12
ΔG_GB_	87.97 ± 3.80
ΔG_SA_	−6.54 ± 0.36
ΔG_bind_	−28.92 ± 3.26

## Data Availability

The original contributions presented in the study are included in the article, further inquiries can be directed to the corresponding authors.
